# Cross-Mapping Events in miRNAs Reveal Potential miRNA-Mimics and Evolutionary Implications

**DOI:** 10.1371/journal.pone.0020517

**Published:** 2011-05-26

**Authors:** Li Guo, Tingming Liang, Wanjun Gu, Yuming Xu, Yunfei Bai, Zuhong Lu

**Affiliations:** 1 State Key Laboratory of Bioelectronics, School of Biological Science and Medical Engineering, Southeast University, Nanjing, China; 2 Jiangsu Key Laboratory for Biodiversity & Biotechnology and Jiangsu Key Laboratory for Aquatic Crustacean Diseases, College of Life Sciences, Nanjing Normal University, Nanjing, China; 3 Key Laboratory of Child Development and Learning Science of Ministry of Education, Southeast University, Nanjing, China; Virginia Tech Virginia, United States of America

## Abstract

MicroRNAs (miRNAs) have important roles in various biological processes. miRNA cross-mapping is a prevalent phenomenon where miRNA sequence originating from one genomic region is mapped to another location. To have a better understanding of this phenomenon in the human genome, we performed a detailed analysis in this paper using public miRNA high-throughput sequencing data and all known human miRNAs. We observed widespread cross-mapping events between miRNA precursors (pre-miRNAs), other non-coding RNAs (ncRNAs) and the opposite strands of pre-miRNAs by analyzing the high-throughput sequencing data. Computational analysis on all known human miRNAs also confirmed that many of them could be involved in cross-mapping events. The processing or decay of both ncRNAs and pre-miRNA opposite strand transcripts may contribute to miRNA enrichment, although some might be miRNA-mimics due to miRNA mis-annotation. Comparing to canonical miRNAs, miRNAs involved in cross-mapping events between pre-miRNAs and other ncRNAs normally had shorter lengths (17–19 nt), lower prediction scores and were classified as pseudo miRNA precursors. Notably, 4.9% of all human miRNAs could be accurately mapped to the opposite strands of pre-miRNAs, which showed that both strands of the same genomic region had the potential to produce mature miRNAs and simultaneously implied some potential miRNA precursors. We proposed that the cross-mapping events are more complex than we previously thought. Sequence similarity between other ncRNAs and pre-miRNAs and the specific stem-loop structures of pre-miRNAs may provide evolutionary implications.

## Introduction

MicroRNAs (miRNAs) are small non-coding RNAs with a length of approximately 22 nucleotides (nt), which play important roles in various biological processes, including various pathophysiological conditions [1], [2], [3], [4], [5]. miRNAs in animals start as primary miRNA transcripts, which are then recognized and processed by the nuclear RNase, Drosha [6]. After processing, miRNA transcripts are turned into miRNA hairpin precursors (pre-miRNA) with stem-loop structures. The hairpin structure is then transported to the cytoplasm, where the RNase, Dicer, cleaves the pre-miRNA into a miRNA/miRNA* duplex [7]. In the biogenesis of miRNA, one strand of the duplex, termed as mature or active miRNA, is then loaded into Ago protein to post-transcriptionally regulate target mRNA. The other strand, known as the inactive strand or miRNA*, is thought to be degraded [8]. However, accumulating evidence has suggested that miRNA* can be loaded into Ago2 protein and contributes to regulate mRNAs as a potential regulatory molecule [9], [10], [11], [12], [13], [14].

To elucidate the potential role of miRNAs in biological processes, it is pivotal to accurately profile the entire miRNA repertoire and compare miRNAomics between different samples. Recently, high-throughput sequencing technologies have been widely applied in identifying and profiling miRNAs at an unprecedented scale with high sensitivity and accuracy. For example, lightly expressed miRNAs can be well profiled in high-throughput miRNA sequencing. IsomiRs, multiple miRNA variants with end sequence variations, have been observed in miRNA deep sequencing datasets from various species [15], [16], [17], [18]. Further studies have suggested that these isomiRs were produced by imprecise and alternative cleavage by Drosha and Dicer during pre-miRNA processing, which may broaden the miRNA-associated regulatory network especially for isomiRs with new “seed sequences” [17], [19].

Although high-throughput sequencing has accelerated our understanding of miRNA biogenesis and function, there are still some issues to be addressed in the analysis of high-throughput miRNA sequencing data. Recent analyses have found a widespread phenomenon of multi-mapping or cross-mapping in deep sequencing datasets of small RNAs [20], [21], [22], [23]. Short reads from high-throughput sequencing can be mapped to multiple loci with an equal number of mismatches in the genome, especially when small RNAs are from multi-copy miRNA precursors and homologous miRNA genes. Generally, the occurrences of such cross-mapping or multi-mapping short reads are randomly assigned to one of the mapped locations, or divided equally among possible locations, or discarded in counting [17], [18], [22], [24], [25]. In a previous study, we proposed that multi-mapping might cause miRNA-mimics and have potential evolutionary implications. A recent study also showed the tRNA-miRNA mimicry in the miRBase database [26]. To better understand the potential relationship between cross-mapping and miRNA mis-annotations, we performed a detailed analysis of cross-mapping events using public deep sequencing data from a human placenta. Due to the involvement of multiple isomiRs with various 5′ and/or 3′ ends and length distributions, we further analyzed the multiple-mapping events in known human miRNAs. According to cross-mapping events in miRNAs through analyzing deep sequencing data, we addressed two questions in this paper: 1) Are there any potential cross-mapping events between known human miRNA precursors and other non-coding RNAs (ncRNAs) such as rRNA, tRNA, snoRNA and snRNA, etc.? 2) Are there any potential cross-mapping events between human miRNA precursors and their opposite strands? If so, these interesting cross-mapping events may provide information about miRNA biogenesis and have evolutionary implications.

## Results

### Widespread cross-mapping events through analyzing high-throughput sequencing data

**Figure 1:**
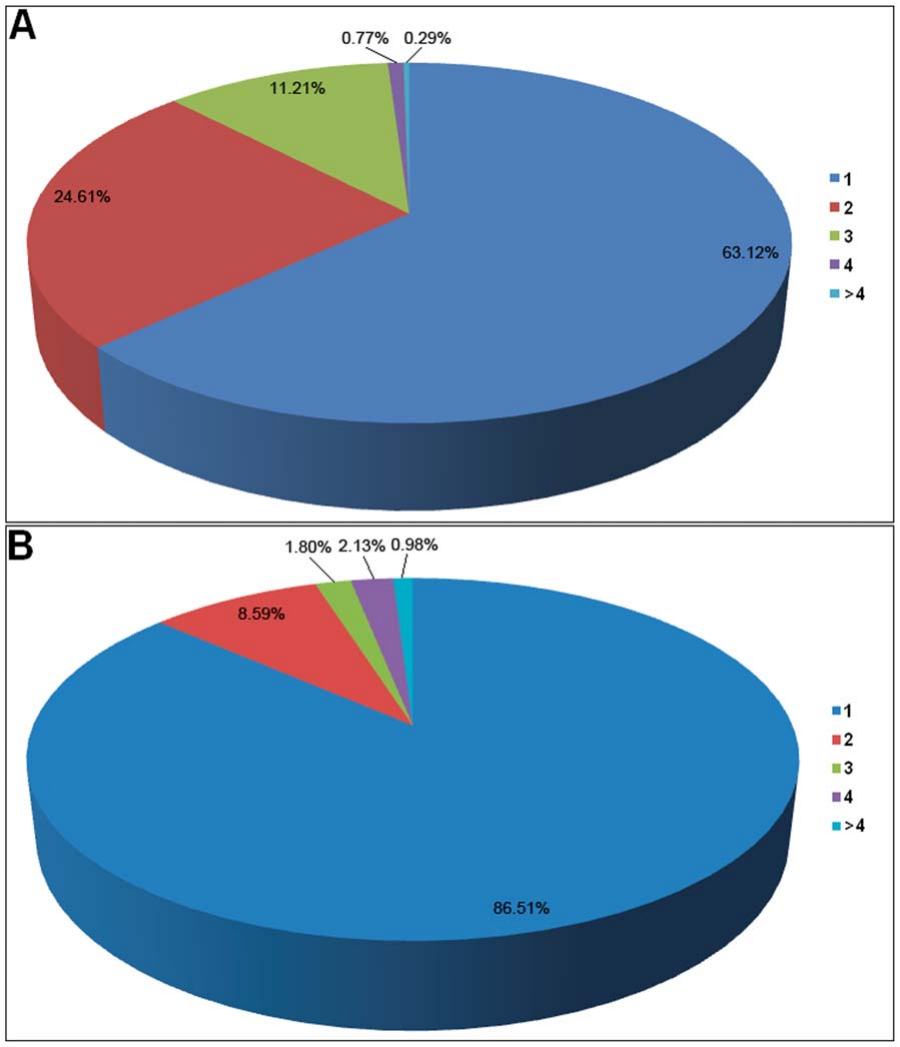
Pie chart illustrating the frequency of cross-mapping. The legends at the right show the number of mapping loci. (A) Frequency of cross-mapping based on known human miRNA precursors through the analysis of high-throughput sequencing data. 36.88% of sequencing reads are simultaneously mapped to two or more different miRNA precursors, with some mapped to over 10 candidate loci. (B) Frequency of cross-mapping based on accurate alignments of known human miRNAs and precursors. Most miRNAs (86.51%) are found in corresponding miRNA precursors; but, 165 miRNAs were detected through the phenomenon of multiple mapping events between different pre-miRNAs. Although multicopy miRNA precursors and homologous miRNA genes contribute to cross-mapping, the fact that miRNAs can be located on opposite strands of pre-miRNAs is also an important factor.

**Figure 2:**
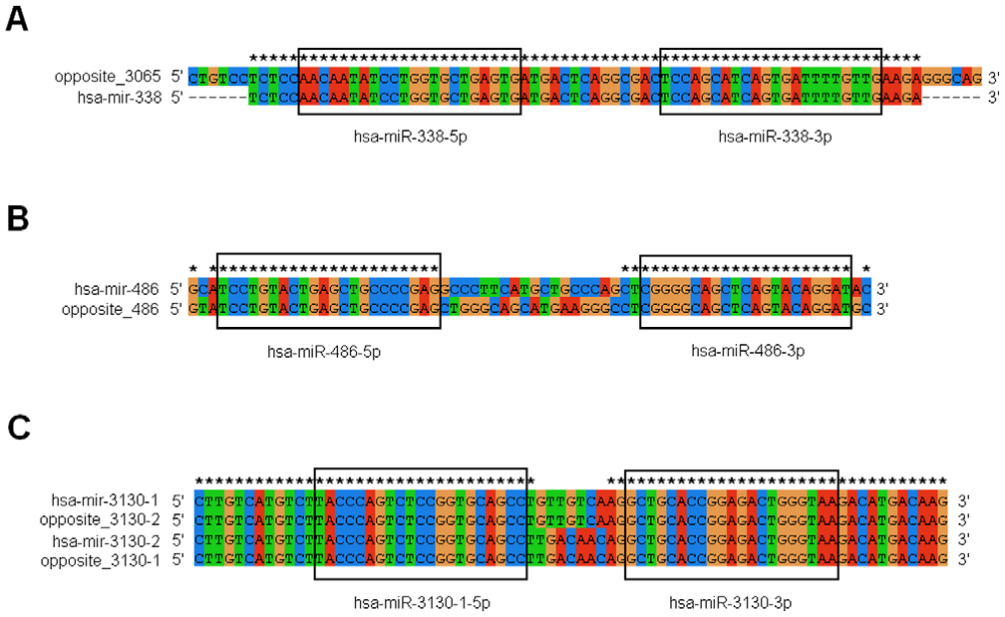
The phenomenon of cross-mapping miRNAs between pre-miRNAs and opposite strands of pre-miRNAs. Sequences in box indicate miRNA sequences. (A) Hsa-miR-338-5p and hsa-miR-338-3p are produced by hsa-mir-338, but they also can accurately map to reverse complementary strand of hsa-mir-3065. (B) Hsa-miR-486-5p and hsa-miR-486-3p can accurately map to hsa-mir-486 and its own opposite strand. (C) Multicopy precursors of hsa-miR-3130 are sense and antisense strands in the same genomic region.

To analyze high-throughput miRNA sequencing data, we first filtered the sequencing reads that could be mapped to other ncRNAs. The remaining reads were then aligned to known human miRNA precursors for identification. Theoretically, filtered reads (those mapped to other ncRNAs) could not be simultaneously mapped to known pre-miRNAs. However, we found that 0.26% of filtered reads were mapped to human pre-miRNAs. This indicated that cross-mapping events could occur between some pre-miRNAs and other ncRNAs due to their high sequence similarity. We also detected cross-mapping events among different pre-miRNAs. 36.88% of sequencing reads could be mapped to more than one (even more than 10 in some cases) human pre-miRNA (Figure 1A). The cross-mapping events were preferentially observed among multi-copy pre-miRNAs and homologous miRNA genes (for example, hsa-miR-26a could be produced by multi-copy precursors of hsa-mir-26a-1 and hsa-mir-26a-2; hsa-mir-26a-1, hsa-mir-26a-2 and hsa-mir-26b were homologous miRNA genes). Interestingly, we observed another 9.91% of reads mapped to the opposite strands of pre-miRNAs (see examples in Figure 2). In some cases, short reads were mapped to one specific pre-miRNA and its opposite strand (Figure 2B).

### Cross-mapping events between pre-miRNAs and other ncRNAs

**Figure 3:**
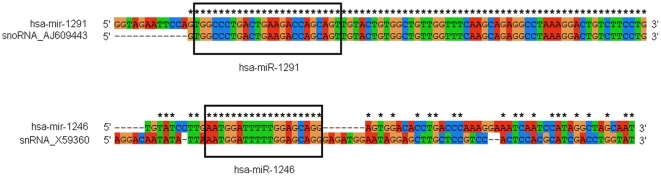
Diversity of sequence similarity between miRNA precursors and other ncRNAs. Boxed sequences indicate common regions (which also is the miRNA sequence) of miRNA precursors and other ncRNAs. Although miRNAs simultaneously map to their precursors and other ncRNAs accurately, miRNA precursors and other ncRNAs show various divergence patterns. They may have consensus sequences (hsa-mir-1291 and snoRNA_AJ609443) or show large divergences (hsa-mir-1246 and snRNA_X59360).

Based on the widespread occurrence of cross-mapping events between miRNA precursors and other ncRNAs in high-throughput sequencing data, we further performed a systematic analysis on all known human miRNAs to identify their potential relationships. Seven human miRNAs (hsa-miR-1291, hsa-miR-1246, hsa-miR-1248, hsa-miR-1274b, hsa-miR-1973, hsa-miR-4284 and hsa-miR-3656) could be accurately mapped to other ncRNAs, which included snoRNAs, snRNAs, tRNAs and rRNAs (Table 1). However, the similarity between their pre-miRNAs and ncRNAs were quite different. For example, hsa-mir-1291 shared a consensus sequence with snoRNA_AJ609443 (Figure 3A). But, the similarity between hsa-mir-1246 and snRNA_X59360 was fairly low (Figure 3B). If mismatches were allowed, more human miRNAs would be involved in cross-mapping events between their pre-miRNAs and other ncRNAs (Table 1). Although sequence similarities were observed between pre-miRNAs and other ncRNAs, they were always located in different genomic regions (Table 1). In general, the sequence similarity between them was low except in the mature miRNA region (Table 1 and Figure 3B). miRNAs that tended to be involved in cross-mapping events with other ncRNAs had lengths ranging from 17 to 27 nt. Most of them were in the range of 17 to19 nt, which is significantly shorter than a typical miRNA (21–24 nt). Their precursors might be pseudo miRNA genes, or were not pre-miRNA-like hairpins or had lower scores according to the miPred web server [27] (Table 1).

**Table 1:** Known human miRNAs simultaneously mapped to other ncRNAs.

MiPred score (%)	miRNA	Length (nt)	Mismatch	ncRNA (No.)	Same?
73.1	miR-1291	24	0	snoRNA (AJ609443)	Yes
61.2 (Pseudo)	miR-1246	19	0	snRNA (X59360)	No
70.9	miR-1248	27	0	snoRNA (AM055745)	Yes
71.0	miR-1274b	17	0	tRNA (U00939)	No
Not_hairpin	miR-1973	19	0	rRNA (DQ304955)	No
73.6 (Pseudo)	miR-4284	18	0	tRNA (AP008562)	No
61.7 (Pseudo)	miR-3656	17	0	rRNA (HSU13369)	No
66.7	miR-1290	19	1	snRNA (HUMUG21)	No
62.1 (Pseudo)	miR-1274a	18	1	tRNA (U00939)	No
Not_hairpin	miR-1322	19	1	snoRNA (AC011292)	No
57.9	miR-3182	17	1	tRNA (X17513)	No
66.6 (Pseudo)	miR-3196	18	1	rRNA (HUMRGM)	No

“Not_hairpin” indicates that the pre-miRNA (for example, hsa-mir-1973) is not a pre-miRNA-like hairpin according to the miPred web server; “Pseudo” indicates that the pre-miRNA is predicated as a pseudo miRNA precursor; “Same?” indicates whether the miRNA precursors and other ncRNAs have the same sequence.

### Cross-mapping events between pre-miRNAs and opposite strands of pre-miRNAs

Other than multiple mapping events between miRNA precursors and other ncRNAs, we also found many miRNAs could be accurately mapped to different miRNA precursors including opposite strands of known pre-miRNAs (Figure 2). 13.49% of all miRNAs were found to have more than one pre-miRNA candidate (Figure 1B). Notably, some miRNAs could have more than four possible pre-miRNA candidates. Since miRNAs are prone to occur in multi-copy miRNA precursors and gene families, it is understandable that cross-mapping events could happen among multi-copy pre-miRNAs and homologous miRNA genes. But, what induces the wide cross-mapping occurrence between pre-miRNAs and their opposite strands? In total, there were 60 (4.9% of all known human miRNAs) miRNAs that could be accurately mapped to opposite strands of known pre-miRNAs (Figure 2 and Table S1). Among these, 18 miRNAs could be specifically mapped to both strands of their own pre-miRNAs (Figure 2B, Figure 2C and Table S1). Some miRNAs can even have both miRNA and its corresponding miRNA* (hsa-miR-#-5p or hsa-miR-#-3p) simultaneously mapped to the opposite strands of their own pre-miRNAs (Figure 2B and Figure 2C). Compared with typical miRNA precursors, pre-miRNAs of these miRNAs always included stem-loop structures with complete complementary miRNA regions (Figure S1). Their opposite strands also always had higher scores when we did potential miRNA precursor predictions using miPred. Some of the opposite strands can even have higher miPred scores than the default pre-miRNAs (Table S1). Moreover, we found almost all of these pre-miRNAs opposite strands were annotated as non-coding sequences in the genome.

Interestingly, cross-mapping of miRNAs also could be detected between miRNA precursors and opposite strands of different pre-miRNAs. For example, both hsa-miR-338-5p and hsa-miR-338-3p could be accurately mapped to the opposite strand of hsa-mir-3065 (Figure 2A). These two miRNA genes shared consensus sequences, in which hsa-mir-338 was a part of the opposite strand of hsa-mir-3065 (Figure 2A). In fact, these two miRNAs were generated from sense and antisense strands of the same genomic location according to the miRBase annotation (hsa-mir-338: chromosome 17 (−): 79,099,683–79,099,749; and hsa-mir-3065: chromosome 17 (+): 79,099,677–79,099,755). We confirmed that each of these pairwise miRNA precursors were located in the same genomic region (Table S1). The results showed that sense and antisense strands in the same genomic region could generate different miRNAs. Furthermore, we found that multi-copy precursors of some miRNAs were actually the sense and antisense strands of a specific genomic region, which could yield the same mature miRNA (for example, hsa-mir-3130-1 and hsa-mir-3130-2, Figure 2C and Table S1).

## Discussion

### Possible miRNA-mimics due to mis-annotations

In analyzing high-throughput sequencing dataset, we found that some short reads could be simultaneously mapped to miRNA precursor sequences and other ncRNAs. Cross-mapping indicated common sequences or high sequence similarities between miRNA precursors and some ncRNAs. This cross-mapping phenomenon was also widely observed among miRNA precursors, especially for the multi-copy precursors and homologous miRNA genes. Notably, many opposite strands of pre-miRNAs could also be involved in cross-mapping, as found in 9.91% of sequencing reads detected. Some miRNA could be mapped to the opposite strand of its own or other unrelated pre-miRNAs (Figure 2). All of these interesting cross-mapping events demonstrated the complexity of miRNA origins, especially when we considered the unavoidable sequencing errors inherent to deep sequencing data analysis. Tolerant mismatches in miRNA sequencing data analysis increased the complexity of cross-mapping. Many short reads could be mapped to multiple genomic locations with the same number of mismatches, but the error profiles could be totally different [23]. Multiple miRNAs with various 5′ and/or 3′ ends and lengths could also partly contribute to the widespread phenomenon of multiple mapping, even though some short RNAs with lengths less than 16 nt were removed from the analysis. Therefore, cross-mapping events, especially those with exact matches, might be false positive miRNAs or miRNA-mimics. A recent paper also showed that miRNA-mimics occurred in the miRBase database based on tRNA-miRNA mimicry due to miRNA mis-annotations [26].

We then asked what this cross-mapping phenomenon would reveal when all human miRNAs were taken into consideration. We performed a comprehensive analysis based on all known human miRNAs, pre-miRNAs and other ncRNAs. As we expected, some miRNAs could be simultaneously mapped to other ncRNAs including rRNAs, tRNAs, snoRNAs and snRNAs (Table 1). The cross-mapping events between pre-miRNAs and other ncRNAs indicated the presence of consensus sequences or common regions (Figure 3). Most of them were located at different genomic loci and showed large sequence divergence except at the common regions (Table 1 and Figure 3B). The common sequence regions of ncRNAs could also yield RNA fragments with the same sequence as miRNAs generated through RNA decay or processing mechanisms. Therefore, it is difficult to distinguish miRNA sequences from those fragments originating from other ncRNAs. Indeed, these miRNAs could also be false positive miRNA-mimics. For example, the hsa-miR-1274b sequence may be small RNA fragments produced by tRNA processing or decay, which will not be actual miRNAs produced by hsa-mir-1274b [26]. Similarly, those miRNAs involved in cross-mapping events, such as hsa-miR-1246 and hsa-miR-4284 in Table 1 (their pre-miRNAs were classified as pseudo miRNA precursors according to the miPred web server), could also be miRNA-mimics that may be by-products of other ncRNAs. More importantly, we found the lengths of these miRNAs were outside the range of typical miRNAs. Most of them were shorter (17–19 nt) than a typical miRNA (∼22 nt) (Table 1). Furthermore, the cross-mapping events between pre-miRNAs and other ncRNAs were also found in mouse and rat miRNAs (data not shown). These miRNAs may be mimics caused by the mis-annotation in the miRBase database as shown in past studies. We caution that these miRNAs should be further validated, especially for those miRNAs predicted by computational methods.

### Sense/antisense miRNAs in the same genomic region

Analysis all known human miRNAs found that 4.9% could be accurately mapped to opposite strands of known miRNA precursors, which could be their own pre-miRNAs or some other pre-miRNAs (Figure 2 and Table S1). Products from the 5′ and 3′arms of the same pre-miRNA could be accurately mapped to the opposite strand of another pre-miRNA. This phenomenon showed that both strands of specific genomic region could be transcribed to generate the same mature miRNAs (for example, hsa-mir-3130-1 and hsa-mir-3130-2, Figure 2C) or different mature miRNAs (for example, hsa-mir-338 and hsa-mir-3065, Figure 2A). Therefore, sense and antisense transcription of specific genomic location could contribute to the miRNA repertoire. The miRNA precursor that is the exact reverse complement of another pre-miRNA was previously considered to be a miRNA-mimic due to miRNA mis-annotations. For example, the dead miRNA entry of hsa-mir-104 (miRNA accession: MI0000110) was updated because hsa-mir-104 is an exact reverse complement of hsa-mir-21 (miRBase database, http://www.mirbase.org/cgi-bin/mirna_entry.pl?acc=MI0000110) [28]. However, we found many different pre-miRNAs that were sense and antisense strands from the same genomic location. Although these miRNAs had the potential to bind complementarily to each other, they often generated different mature miRNAs (Figure S2) and showed inconsistent expression levels (Figure S3). Taken together, we proposed that other mature miRNAs could be generated from the reverse complement of pre-miRNAs. Since the miRNAs generated from sense and antisense strands of one specific genomic region could complementarily bind to each other, they will restrict the transcription process of one another [29], [30], [31]. This binding could be a potential regulatory mechanism among miRNAs, which could play an important role in the dynamics of miRNA profiles.

Moreover, we found some miRNAs could be accurately mapped to opposite strands of their own pre-miRNAs (Figure 2B and Table S1). This interesting phenomenon could be more common if there were more miRNA* sequences for analysis. For example, we found that hsa-mir-559 and its opposite strand had consensus sequences in 5′ and 3′ arms, respectively. Because many miRNA* sequences have low expression levels, limited miRNA* sequences are discovered and collected in the miRBase database. Accurate cross-mapping of miRNA/miRNA* demonstrated that sense and antisense transcription of pre-miRNA may greatly contribute to miRNA expression profiles. We found that opposite strands of pre-miRNAs also had high miPred scores as potential miRNA precursors. Some of them have even higher miPred scores than the default miRNA precursors (Table S1). More importantly, almost all of the opposite strands of pre-miRNAs were annotated as non-coding sequences. Based on these results, we inferred that the opposite strands of these pre-miRNAs could be potential pre-miRNAs and also generate mature miRNAs (termed potential multi-copy miRNA precursors). Indeed, some validated multi-copy miRNA precursors were generated from the sense and antisense strands of the same genomic region (Figure 2C). More systematic analysis is needed to look into this interesting phenomenon and validate these possible multi-copy miRNA precursors.

### Potential evolutionary implications

When miRNA cross-mapping occurs, the target reference sequences, pre-miRNAs and other ncRNAs, showed different levels of similarity in consensus sequences or large sequence divergences except in common regions (Figure 3). If mismatches were allowed, more miRNAs would be involved in the cross-mapping phenomenon between pre-miRNAs and other ncRNAs (Table 1). Despite the fact that miRNAs with accurate cross-mapping may be miRNA-mimics, we can not ignore the possibility that cross-sequences or similar sequences may have evolutionary implications. Based on their sequence similarity, some miRNA genes might be directly or indirectly derived from other ncRNAs, such as tRNAs, rRNAs, snoRNAs or snRNAs. Perhaps it provides an evolutionary relationship between different ncRNAs as indicated by miRNAs that are very well conserved phylogenetically across larger evolutionary distances as seen in vertebrates and fruit flies [32], [33], [34]. We also found that some miRNAs could be accurately mapped to opposite strands of their own pre-miRNAs (Figure 2 and Table S1). It is well known that miRNA precursors can form a stem-loop structure with the miRNA located in the 5′ or 3′arm. These special pre-miRNAs showed stem-loop structures with complete complementary miRNA regions (Figure S1). The strand symmetry and stem-loop structures with incomplete or complete complementary miRNA regions might provide some evolutionary implications and should be taken into account in miRNA biogenesis analysis.

## Materials and Methods

Public high-throughput sequencing dataset of small RNAs from human placental sample generated from the SOLiD™ System (ABI, Life Technologies) were obtained (http://SOLiDsoftwaretools.com/gf/project/srna/) and analyzed using the SOLiD miRNA analysis pipeline. The pipeline includes three major steps: 1) Filter known human non-coding RNAs, such as tRNAs, rRNAs, snoRNA, snRNAs, etc. All ncRNAs were collected from GenBank (http://www.ncbi.nlm.nih.gov/genbank/). 2) Detect known miRNAs by aligning them to human miRNA precursor sequences in the miRBase database (Release 16.0, http://www.mirbase.org/). 3) Discover novel miRNAs by mapping unaligned reads using the human genome sequence as a reference. To detect known miRNAs, the short reads were first aligned to miRNA precursors using a seed 16-mer with allowing only 2 mismatches. For those reads matching the seed sequence, full-length (35 nt for SOLiD reads) alignments were performed with 4 mismatches allowed. Short reads were removed from the analysis if their lengths are less than 16 nt after 3′ adaptor sequences were discarded. Only short RNAs that could be mapped to known miRNA precursors were used for further analysis of multiple isomiRs. Due to shorter sizes and tolerant mismatches, the phenomenon of multiple mapping or cross-mapping could be detected with an equal number of mismatches.

To elucidate potential relationships between miRNA precursors, other ncRNAs, and pre-miRNAs and their opposite strands, we also performed a comprehensive analysis using all known human miRNAs and their precursor sequences in the miRBase database. All known human miRNAs were aligned to known miRNA precursors and other ncRNAs using the Bowtie (version 0.12.7) [35]. Only those miRNAs that could be mapped to the sense strands of other ncRNAs and pre-miRNAs were considered in further analyses. To have a detailed view of the opposite strands of pre-miRNAs, we also collected those miRNAs that could be accurately mapped to the reverse complementary sequences of human miRNA precursors. miPred scores of opposite strands and corresponding pre-miRNAs were estimated using the miPred web server [27]. Multiple sequence alignments of miRNA precursors and other ncRNAs were performed using the Clustal X (version 2.0) [36].

## Supporting Information

Figure S1 **Human miRNA precursors' stem-loop structures from the miRBase database.** Sequences with a pink background indicate miRNA and miRNA*. (A) Generally, miRNA precursors can form stem-loop structures with some incomplete complementary miRNA regions. (B) If miRNAs can simultaneously map to sense and antisense strands of their own pre-miRNAs, these special precursors may form complete complementary miRNA regions.
(TIF)

Figure S2 **Different miRNAs from sense and antisense strands in the same genomic region can complementarily bind to each other.** The binding events maybe provide potential regulation among different miRNAs.
(TIF)

Figure S3 **Inconsistent expression levels of miRNAs (sense/antisense miRNAs) from the same genomic region.** Pairwise miRNAs are generated from the same genomic region; however, their expression levels show significant differences. Generally, one miRNA shows a higher percentage (more than 90%).
(TIF)

Table S1 **Accurate cross-mapping of miRNAs with pre-miRNAs and the opposite strands of pre-miRNAs.**
(DOC)
