# Supplementary figures and images for: Cross-Mapping Events in miRNAs Reveal Potential miRNA-Mimics and Evolutionary Implications
Source: PLoS One. 2011 May 26;6(5):e20517. doi: 10.1371/journal.pone.0020517 (PMC3102724; doi:10.1371/journal.pone.0020517)

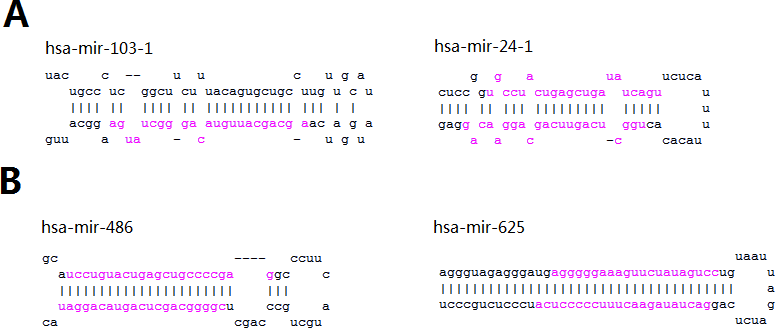

Supplement: Figure S1 — Human miRNA precursors' stem-loop structures from the miRBase database. Sequences with a pink background indicate miRNA and miRNA*. (A) Generally, miRNA precursors can form stem-loop structures with some incomplete complementary miRNA regions. (B) If miRNAs can simultaneously map to sense and antisense strands of their own pre-miRNAs, these special precursors may form complete complementary miRNA regions. (TIF) [file pone.0020517.s001.tif]

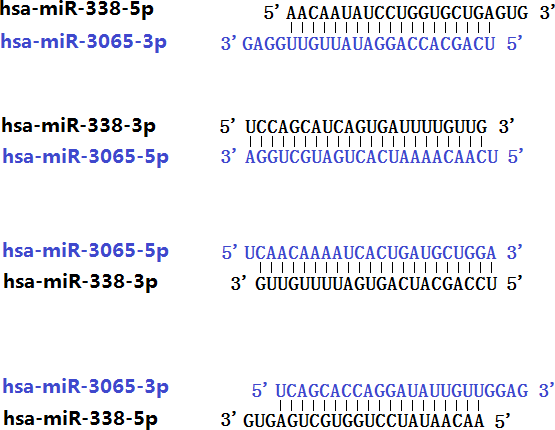

Supplement: Figure S2 — Different miRNAs from sense and antisense strands in the same genomic region can complementarily bind to each other. The binding events maybe provide potential regulation among different miRNAs. (TIF) [file pone.0020517.s002.tif]

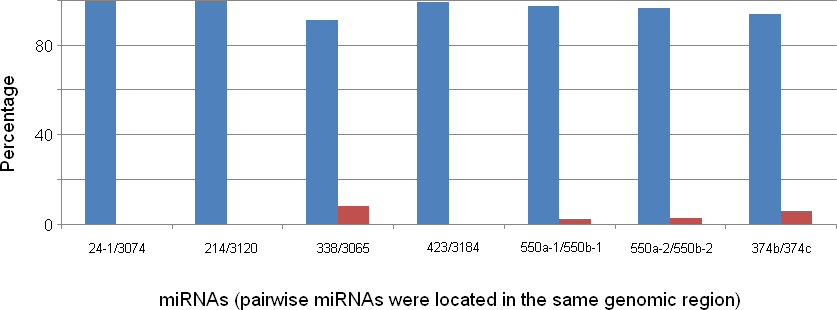

Supplement: Figure S3 — Inconsistent expression levels of miRNAs (sense/antisense miRNAs) from the same genomic region. Pairwise miRNAs are generated from the same genomic region; however, their expression levels show significant differences. Generally, one miRNA shows a higher percentage (more than 90%). (TIF) [file pone.0020517.s003.tif]
