# Supplementary material for: Cross-Mapping Events in miRNAs Reveal Potential miRNA-Mimics and Evolutionary Implications
Source: PLoS One. 2011 May 26;6(5):e20517. doi: 10.1371/journal.pone.0020517 (PMC3102724; doi:10.1371/journal.pone.0020517)
Supplement: Table S1 — Accurate cross-mapping of miRNAs with pre-miRNAs and the opposite strands of pre-miRNAs. (DOC) [file pone.0020517.s004.doc]

**Table S1.** **Accurate cross-mapping of miRNAs with pre-miRNAs and the opposite strands of pre-miRNAs.**

| **MiPred score (%)** | **miRNA** | **Opposite strand of pre-miRNA** | **MiPred score (%)** |
| --- | --- | --- | --- |
| 73.9 | miR-24-1* | oppo-mir-3074 | The same region |
| 73.9 | miR-24-1 |  |  |
| 75.3 | miR-103-2* | oppo-mir-103-2-as | The same region |
| 87.2 | miR-103-1 | oppo-mir-103-1-as | The same region |
| 75.3 | miR-103-2 | oppo-mir-103-2-as | The same region |
| No | miR-103-1-as | oppo-mir-103-1 | The same region |
| 68.1 (pseudo) | miR-103-2-as | oppo-mir-103-2 | The same region |
| 76.5 | miR-214* | oppo-mir-3120 | The same region |
|  | miR-214 |  |  |
| 71.2 | miR-338-5p | oppo-mir-3065 | The same region |
|  | miR-338-3p |  |  |
| 85.1 | miR-374b | oppo-mir-374c | The same region |
|  | miR-374b* |  |  |
| 69.5 | miR-374c | oppo-mir-374b | The same region |
| 72.6 | miR-423-5p | oppo-mir-3184 | The same region |
|  | miR-423-3p |  |  |
| 73.3 | miR-486-5p | oppo-mir-486 | 77.7 |
|  | miR-486-3p |  |  |
| 79.6 | miR-548aa-1 | oppo-mir-548d-1 | The same region |
| 75.3 | miR-548aa-2 | oppo-mir-548d-2 | The same region |
| 80.5 | miR-548c-5p | oppo-mir-548z | The same region |
|  | miR-548c-3p |  |  |
| 82.6 | miR-548d-1-5p | oppo-mir-548aa-1 | The same region |
|  | miR-548d-1-3p |  |  |
| 79.5 | miR-548d-2-5p | oppo-mir-548aa-2 | The same region |
|  | miR-548d-2-3p |  |  |
| 72.7 | miR-548z | oppo-mir-548c | The same region |
| 75.2 | miR-549 | oppo-mir-549 | 78.6 |
| 83.4 | miR-550a-1 | oppo-mir-550b-1 | The same region |
|  | miR-550a*-1 |  |  |
| 81.7 | miR-550a-2 | oppo-mir-550b-2 | The same region |
|  | miR-550a*-2 |  |  |
| 64.8 | miR-550b-1 | oppo-mir-550a-1 | The same region |
| 64.0 | miR-550b-2 | oppo-mir-550a-2 | The same region |
| 79.7 | miR-559 | oppo-mir-559 | 77.0 |
| 78.5 | miR-610 | oppo-mir-610 | 88.0 |
| 80.9 | miR-625 | oppo-mir-625 | 80.4 |
|  | miR-625* |  |  |
| 71.2 | miR-642a | oppo-mir-642b | The same region |
| 74.6 | miR-642b | oppo-mir-642a | The same region |
| 77.2 | miR-1258 | oppo-mir-1258 | 74.4 |
| 68.7 | miR-3065-5p  miR-3065-3p | oppo-mir-338 | The same region |
| 67.2 | miR-3074 | oppo-mir-24-1 | The same region |
| 79.6 | miR-3116-1^a^ | oppo-mir-3116-2 | The same region |
| 73.3 | miR-3116-2^a^ | oppo-mir-3116-1 | The same region |
| 81.0 | miR-3119-1^a^ | oppo-mir-3119-2 | The same region |
| 75.4 | miR-3119-2^a^ | oppo-mir-3119-1 | The same region |
| 77.3 | miR-3120 | oppo-mir-214 | The same region |
| 78.6 | miR-3130-1-5p^a^ | oppo-mir-3130-2 | The same region |
|  | miR-3130-1-3p^a^ |  |  |
| 81.4 | miR-3130-2-5p^a^ | oppo-mir-3130-1 | The same region |
|  | miR-3130-2-3p^a^ |  |  |
| 77.6 | miR-3150 | oppo-mir-3150b | The same region |
| 75.0 | miR-3150b | oppo-mir-3150 | The same region |
| 81.2 | miR-3158-1^a^ | oppo-mir-3158-2 | The same region |
| 74.1 | miR-3158-2^a^ | oppo-mir-3158-1 | The same region |
| 79.1 | miR-3160-1^a^ | oppo-mir-3160-2 | The same region |
| 79.4 | miR-3160-2^a^ | oppo-mir-3160-1 | The same region |
| 68.5 | miR-3184 | oppo-mir-423 | The same region |
| 70.3 | miR-3190 | oppo-mir-3191 | The same region |
| 76.1 | miR-3191 | oppo-mir-3190 | The same region |
| 81.6 | miR-3199-1^a^ | oppo-mir-3199-2 | The same region |
| 84.8 | miR-3199-2^a^ | oppo-mir-3199-1 | The same region |
| 79.4 | miR-3202-1^a^ | oppo-mir-3202-2 | The same region |
| 75.0 | miR-3202-2^a^ | oppo-mir-3202-1 | The same region |
| 74.6 | miR-3622a-5p | oppo-mir-3622b | The same region |
|  | miR-3622a-3p |  |  |
| 72.1 | miR-3622b-5p | oppo-mir-3622a | The same region |
|  | miR-3622b-3p |  |  |
| 83.7 | miR-3688 | oppo-mir-3688 | 80.3 |
| 79.0 | miR-3910-1^a^ | oppo-mir-3910-2 | The same region |
| 79.0 | miR-3910-2^a^ | oppo-mir-3910-1 | The same region |
| 78.7 | miR-3913-1^a^ | oppo-mir-3913-2 | The same region |
| 80.8 | miR-3913-2^a^ | oppo-mir-3913-1 | The same region |
| 77.1 | miR-3914-1^a^ | oppo-mir-3914-2 | The same region |
| 79.0 | miR-3914-2^a^ | oppo-mir-3914-1 | The same region |
| 74.2 | miR-3926-1^a^ | oppo-mir-3926-2 | The same region |
| 68.4 | miR-3926-2^a^ | oppo-mir-3926-1 | The same region |

miRNAs are named according to their pre-miRNA sequences (hsa-miR-24-1 is generated from hsa-mir-24-1). The first column shows miPred scores of miRNA precursors according to the second column. “The same region” shows that pre-miRNAs are located in the same genomic region (+/-). For example, hsa-mir-24-1 and hsa-mir-3074 are factual sense and antisense strands from the same genomic region. ^a^ indicates that multi-copy pre-miRNAs are factual sense and antisense strands and are located in the same region (for example, hsa-mir-3130-1 and hsa-mir-3130-2 ).
